# Depletion and activation of microglia impact metabolic connectivity of the mouse brain

**DOI:** 10.1186/s12974-023-02735-8

**Published:** 2023-02-24

**Authors:** Johannes Gnörich, Anika Reifschneider, Karin Wind, Artem Zatcepin, Sebastian T. Kunte, Philipp Beumers, Laura M. Bartos, Thomas Wiedemann, Maximilian Grosch, Xianyuan Xiang, Maryam K. Fard, Francois Ruch, Georg Werner, Mara Koehler, Luna Slemann, Selina Hummel, Nils Briel, Tanja Blume, Yuan Shi, Gloria Biechele, Leonie Beyer, Florian Eckenweber, Maximilian Scheifele, Peter Bartenstein, Nathalie L. Albert, Jochen Herms, Sabina Tahirovic, Christian Haass, Anja Capell, Sibylle Ziegler, Matthias Brendel

**Affiliations:** 1 Department of Nuclear Medicine, University Hospital, Ludwig-Maximilians-Universität München, Marchioninistrasse 15, 81377 Munich, Germany; 2 German Center for Neurodegenerative Diseases (DZNE), Munich, Germany; 3 Metabolic Biochemistry, Faculty of Medicine, Biomedical Center (BMC), Ludwig-Maximilians-Universität München, Munich, Germany; 4 German Center for Vertigo and Balance Disorders, University Hospital, Ludwig-Maximilians-Universität München, Munich, Germany; 5 CAS Key Laboratory of Brain Connectome and Manipulation, The Brain Cognition and Brain Disease Institute, Shenzhen Institutes of Advanced Technology, Chinese Academy of Sciences, Shenzhen-Hong Kong Institute of Brain Science-Shenzhen Fundamental Research Institutions, Shenzhen, 518055 China; 6 Center for Neuropathology and Prion Research, Ludwig-Maximilians-Universität München, Munich, Germany; 7 Munich Cluster for Systems Neurology (SyNergy), Munich, Germany

**Keywords:** FDG–PET, Neurodegeneration, Microglia, Metabolic connectivity, scRadiotracing

## Abstract

**Aim:**

We aimed to investigate the impact of microglial activity and microglial FDG uptake on metabolic connectivity, since microglial activation states determine FDG–PET alterations. Metabolic connectivity refers to a concept of interacting metabolic brain regions and receives growing interest in approaching complex cerebral metabolic networks in neurodegenerative diseases. However, underlying sources of metabolic connectivity remain to be elucidated.

**Materials and methods:**

We analyzed metabolic networks measured by interregional correlation coefficients (ICCs) of FDG–PET scans in WT mice and in mice with mutations in progranulin (*Grn*) or triggering receptor expressed on myeloid cells 2 (*Trem2*) knockouts (^−/−^) as well as in double mutant *Grn*^−/−^/*Trem2*^−/−^ mice. We selected those rodent models as they represent opposite microglial signatures with disease associated microglia in *Grn*^*−/−*^ mice and microglia locked in a homeostatic state in *Trem2*^*−/−*^ mice*;* however, both resulting in lower glucose uptake of the brain*.* The direct influence of microglia on metabolic networks was further determined by microglia depletion using a CSF1R inhibitor in WT mice at two different ages. Within maps of global mean scaled regional FDG uptake, 24 pre-established volumes of interest were applied and assigned to either cortical or subcortical networks. ICCs of all region pairs were calculated and z-transformed prior to group comparisons. FDG uptake of neurons, microglia, and astrocytes was determined in *Grn*^−/−^ and WT mice via assessment of single cell tracer uptake (scRadiotracing).

**Results:**

Microglia depletion by CSF1R inhibition resulted in a strong decrease of metabolic connectivity defined by decrease of mean cortical ICCs in WT mice at both ages studied (6–7 m; *p* = 0.0148, 9–10 m; *p* = 0.0191), when compared to vehicle-treated age-matched WT mice. *Grn*^−/−^, *Trem2*^−/−^ and *Grn*^*−/−*^*/Trem2*^*−/−*^ mice all displayed reduced FDG–PET signals when compared to WT mice. However, when analyzing metabolic networks, a distinct increase of ICCs was observed in *Grn*^*−/−*^ mice when compared to WT mice in cortical (*p* < 0.0001) and hippocampal (*p* < 0.0001) networks. In contrast, *Trem2*^*−/−*^ mice did not show significant alterations in metabolic connectivity when compared to WT. Furthermore, the increased metabolic connectivity in *Grn*^*−/−*^ mice was completely suppressed in *Grn*^*−/−*^*/Trem2*^*−/−*^ mice. *Grn*^*−/−*^ mice exhibited a severe loss of neuronal FDG uptake (− 61%, *p* < 0.0001) which shifted allocation of cellular brain FDG uptake to microglia (42% in *Grn*^*−/−*^ vs. 22% in WT).

**Conclusions:**

Presence, absence, and activation of microglia have a strong impact on metabolic connectivity of the mouse brain. Enhanced metabolic connectivity is associated with increased microglial FDG allocation.

**Supplementary Information:**

The online version contains supplementary material available at 10.1186/s12974-023-02735-8.

## Highlights

- Microglial activation influences metabolic connectivity.
- Microglial depletion results in distinct decreases of metabolic connectivity.
- Metabolic connectivity increases in progranulin deficient mice.
- Cellular FDG allocation in progranulin deficient mice is shifted to microglia.

**Supplementary Information:**

The online version contains supplementary material available at 10.1186/s12974-023-02735-8.

## Introduction

Frontotemporal dementia (FTD) is the second most common pre-senile neurodegenerative disease after Alzheimer’s disease (AD) which is the most common cause of dementia and age-related neurodegenerative disorders and thus present a critical public health issue. Various studies presented that neurodegenerative diseases such as FTD [29] or AD [58] may lead to severe alterations in brain metabolism detectable with positron emission tomography (PET). Clinical PET imaging with the glucose analog ^18^F-Fluordesoxyglucose (FDG) has become an essential procedure in distinguishing between AD and non-AD dementias [62] as well as in detecting deficits in cerebral glucose metabolism characteristics of neurodegenerative diseases [7]. Although FDG–PET is not perfectly specific, for neurodegenerative disease, it has been a useful clinical biomarker for decades [41] and presents one of the most sensitive functional biomarkers of AD and other neurodegenerative disease in general diseases [27], [58].

The invention of functional magnetic resonance imaging (fMRI) enabled detecting brain inter-regional anatomical and functional networks by comparing regional oxygen consumption [13]. Combining topographies of FDG–PET with fMRI further enhances evaluation of metabolic and neuronal interactions [65]. Current research tends to focus on long-distance effects of brain pathology of interconnected neural systems rather than local neuronal phenomena [14, 59]. Lately, several reviews highlighted different approaches of identifying metabolic [2, 54] and functional connectivity [15]. In addition, to the already established impact in basic science, PET studies involving analyses of metabolic connectivity are of growing clinical importance. In recent studies concerning neurodegenerative diseases, such as dementia [30, 42] or movement disorders [28], functional and cognitive impairment was suggested to be caused by disrupted interactions of connected brain regions directly linked to neuroinflammation [48]. Importantly, metabolic connectivity already showed alterations by disease-related patterns [44, 61] in FTD [37, 60], at prodromal disease stages in Dementia with Lewy bodies (DLB) [28] and AD [55], which indicate its potential usefulness as an early biomarker and, furthermore, addressing the lack of specificity of FDG–PET in differential diagnosis of neurodegenerative disease.

FDG–PET is also widely used in a variety of preclinical models of neurodegenerative diseases [10, 18, 32, 33, 52]. However, a systematic analysis of how various brain cells contribute to metabolic connectivity in neurodegenerative conditions has not been performed yet. This research gap needs to be closed, since glucose is not only taken up by neurons but also by astrocytes [70] and microglia [67].In particular, we observed that glucose uptake of microglia has a strong impact on FDG–PET signals [67]. Thus, we questioned if microglia and their activation status have an impact on brain metabolic connectivity.

First, we used pharmacological microglia depletion in wild-type (WT) mice at two different ages to evaluate changes in metabolic connectivity in the absence of microglia cells. Next, we compared metabolic connectivity in mouse models with opposite microglia phenotypes, to test if microglial activation states impact the metabolic connectome. For this purpose, we analyzed progranulin knockout (*Grn*^−/−^) mice with disease-associated microglia (DAM) [18, 38] and triggering receptor expressed on myeloid cells 2 knockout (*Trem2*^*−/−*^) mice with microglia locked in a homeostatic stage [31, 32, 40] as they both exhibit reduced glucose uptake in FDG–PET [18, 53].

Finally, we used cell sorting after FDG injection to test which cellular alterations drive metabolic connectivity and performed immunoblotting to search for alterations in abundance of microglia in *Grn*^−/−^ and WT mice. All data were analyzed in relation to standardized uptake values (SUV) of FDG–PET, aiming to evaluate the potential of metabolic connectivity as a complementary read-out in mouse models with altered microglial activation.

## Methods

### Experimental setup and study design

All FDG–PET scans used in this study were obtained from previous investigations of our research group or derived from not yet published data, all performed in a standardized setting. The experiments have been approved by the local animal care committee of the Government of Oberbayern (Regierung Oberbayern), overseen by a veterinarian and in compliance with the ARRIVE guidelines and were carried out in accordance with the U.K. Animals (Scientific Procedures) Act, 1986 and associated guidelines, EU Directive 2010/63/EU for animal experiments. Animals were housed in a temperature- and humidity-controlled environment with a 12 h light–dark cycle, with free access to food (Ssniff) and water. Anesthesia during tracer application and PET scanning was induced with 3,0% isoflurane and maintained with isoflurane 1.5% delivered via a mask at 3,5 L/min. The following mice groups were analyzed: WT mice at the age of 6 (*n* = 9) and 9 (*n* = 8) months treated with PLX 5622 at 1200 ppm, vehicle-treated controls of the same age (6 months: *n* = 14; 9 months: *n* = 13) [57, 67], *Grn*^*−/−*^ mice (*n* = 10), *Grn*^*−/−*^*/Trem2*^*−/−*^ mice (*n* = 10), and *Trem2*^*−/−*^ mice (*n* = 13), at an average age of 10.6 ± 1.5 months, together with age-matched WT mice (*n* = 17) [53]. N = 9 *Grn*^*−/−*^ and *n* = 8 WT mice underwent scRadiotracing [5, 67]. A detailed overview of the study groups is provided in Table 1.

**Table 1:** Summary of analyzed data

Mouse model	Age (months)	Animal (*n*)	Scanner	Sex (f/m)	Weight (g)	Injected dose (MBq)
WT with PLX5622 treatment	6–7	9	PET	4/5	29.7 ± 4.6	14.0 ± 1.9
	9–10	8	PET	8/0	24.3 ± 3.3	14.2 ± 2.2
WT with placebo treatment (control)	6–7	14	PET	10/4	25.4 ± 4.0	14.6 ± 2.0
	9–10	13	PET	11/2	24.7 ± 3.1	14.0 ± 2.2
*Grn*^*−/−*^	9–12	10	PET–MRI	10/0	26.9 ± 2.6	15.5 ± 2.5
*Trem2*^*−/−*^	9–12	13	PET–MRI	13/0	28.4 ± 4.7	15.2 ± 2.5
*Grn*^*−/−*^*/Trem2*^*−/−*^	9–12	10	PET–MRI	10/0	26.6 ± 1.3	17.3 ± 1.5
WT	9–12	17	PET–MRI	17/0	27.2 ± 3.1	14.1 ± 2.2

### Image acquisition

PET images were acquired either using a dedicated PET-scanner (Siemens Inveon DPET) or a PET–MRI (3T Mediso nanoScan PET/MR scanner, Mediso Ltd, Hungary) with harmonized acquisition and reconstruction protocols to ensure comparable spatial resolution.

In brief, mice were placed in the aperture of the Siemens Inveon DPET in a four-bed mouse hotel. Static single frame emission recordings were made in the interval 30–60 min after bolus injection of [^18^F]-FDG (in 150 μl saline) to a tail vein, followed by a 15 min transmission scan made using a rotating [^57^Co] point source. Prior to the PET scan no food was administered for ≥ 4 h. The image reconstruction procedure was performed with a three-dimensional ordered subset expectation maximization (OSEM) with 4 iterations and 12 subsets followed by a maximum-a-posteriori (MAP) algorithm with 32 iterations. All data were random, scatter-, attenuation-, and decay-corrected and processed with a zoom factor of 1.0 leading to a final voxel dimension of 0.78 × 0.78 × 0.80 mm^3^. All reconstructions were standardized to ensure comparability of the scans [46].

When scanning with the 3T Mediso nanoScan PET/MR, we used a single-mouse imaging chamber. A 15-min anatomical T1 MR scan was performed at 15 min after [^18^F]-FDG injection (head receive coil, matrix size 96 × 96 × 22, voxel size 0.24 × 0.24 × 0.80 mm^3^, repetition time 677 ms, echo time 28.56 ms, flip angle 90°). PET emission was recorded at 30–60 min p.i.. PET list-mode data were reconstructed using a 3D iterative algorithm (Tera-Tomo 3D, Mediso Ltd, Hungary) with the following parameters: matrix size 55 × 62 × 187 mm^3^, voxel size 0.3 × 0.3 × 0.3 mm^3^, 8 iterations, 6 subsets. Random, scatter, attenuation, and decay corrections were applied. The T1 image was used to create a body-air material map for scatter and attenuation correction.

### Image preprocessing and analysis

**Fig. 1:**
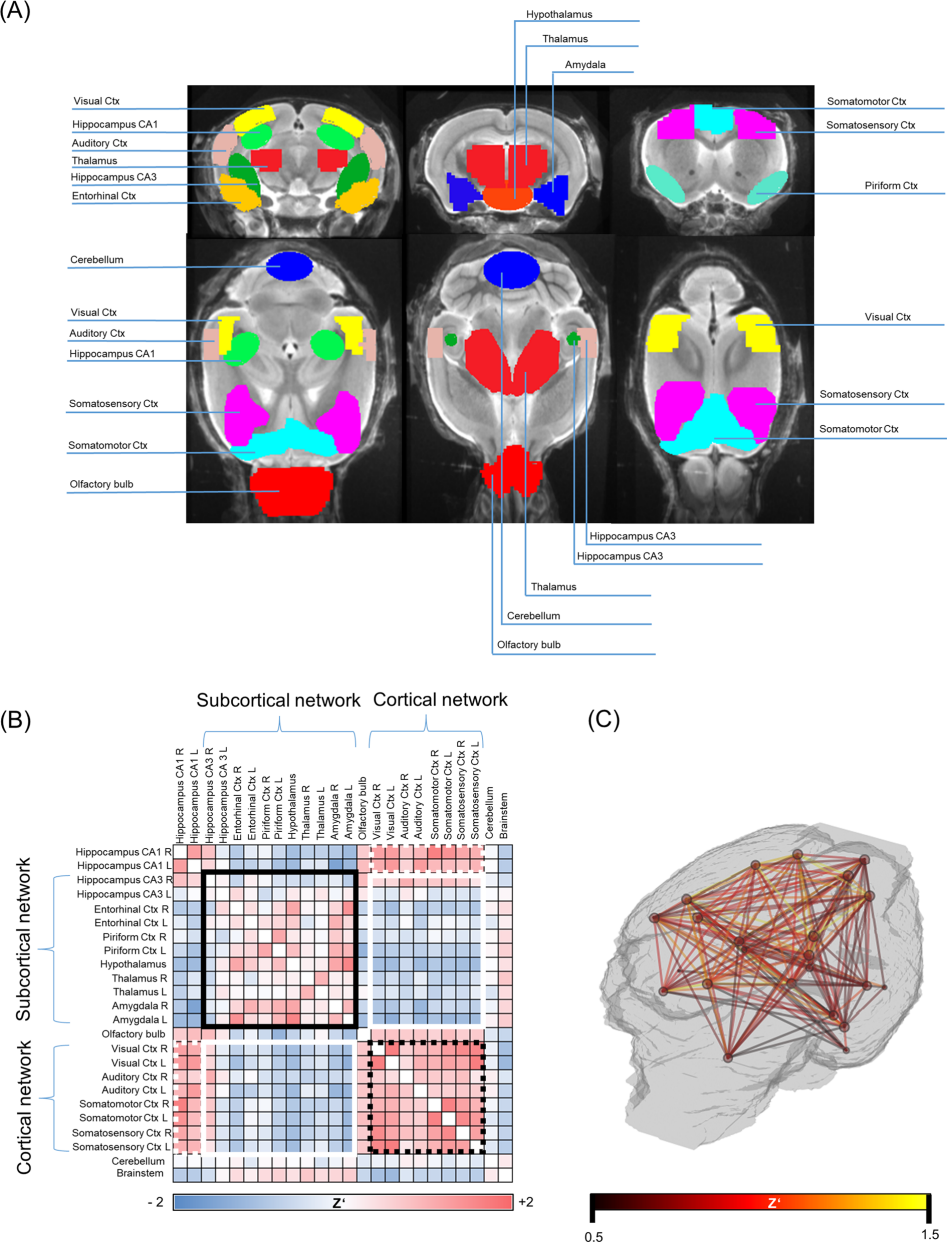
Volume of interest (VOI) definition evaluation and image processing. **A** VOIs representing certain functional brain areas are highlighted in different colors. MRI template depicts the anatomical correlate of the defined brain regions. **B** Exemplary correlation matrix labelled with 24 predefined VOIs. **C** Exemplary 3D mouse brain template displays hot-scaled absolute ICCs exceeding the threshold of *Z*’ > 0.5

FDG–PET preprocessing for all image data was performed by PMOD (V3.5, PMOD technologies, Basel, Switzerland). For PET images derived from the PET–MRI, a Gaussian filter (1.0 mm^3^) was applied for harmonization of the full-width-at-half-maximum, i.e., to achieve similar spatial resolution [53]. Unified spatial normalization (nonlinear warping, 0.6 mm^3^ Gaussian smoothing of the input image, 16 iterations, frequency cutoff 3, no thresholding) of all original FDG–PET images to the same previously established FDG–PET template was performed, to ensure spatial comparability between all different studies. Standardized uptake values (SUVs), the common index of tracer uptake, for target volumes of interest (VOIs) were calculated by scaling to the injected dose and normalizing for body weight (tracer uptake in VOI/(injected activity/mouse weight)). Individual FDG–PET images were intensity-normalized to their global mean to obtain maps of relative regional FDG uptake as standardized uptake value ratios (SUVrs) in 24 cortical and subcortical volumes of interest (VOIs) (Fig. 1A, B). The VOIs were defined on a T1 MRI template (Fig. 1A) in Ma-Benveniste-Mirrione atlas space including anatomical compartments and functional subdivision [36]. All data were analyzed in relation to common SUV and SUVr quantification of FDG–PET, aiming at evaluating the potential of metabolic connectivity as a complementary read-out.

### Mouse brain dissociation

*N* = 9 *Grn*^*−/−*^ and *n* = 8 WT mice underwent scRadiotracing [5, 67]. Adult Brain Dissociation Kit (mouse and rat) (Miltenyi Biotec, 130-107-677) was used for brain dissociation according to the manufacturer's instructions. Adult mouse brains were dissected, briefly washed with Phosphate-buffered saline (PBS), cut into eight pieces, and dissociated with enzyme mix 1 and 2 using gentleMACS™ Octo Dissociator (Miltenyi Biotec, 130-096-427). The dissociated cell suspension was applied to a pre-wet 100 µm Cell Strainer (Falcon, 352360). The cell pellet was resuspended using cold PBS and cold debris removal solution. Cold PBS was gently overlaid on the cell suspension. Centrifuged at 4 °C and 3000×*g* for 10 min with acceleration and deceleration at 5. The two top phases were removed entirely. The cell pellets were collected and resuspended with 1 ml cold red blood cell removal solution followed by 10 min incubation. Cell pellets were collected for neuron isolation via magnetic activated cell sorting (MACS) [5, 67] and microglia and astrocyte isolation via Fluorescence Activated Cell Sorting (FACS).

### Isolation of neurons

Neuronal isolation via MACS was performed from *n* = 5 *Grn*^*−/−*^ and *n* = 5 WT brains. Neuron Isolation Kit, mouse (Miltenyi Biotec, 130-115-390), was used according to the manufacturer's instructions. The prepared cell pellets were resuspended in 80 µl of PBS-0.5% Bovine Serum Albumin (BSA) buffer per 10^7^ total cells. 20 μl of Non-Neuronal Cells Biotin-Antibody Cocktail was added and incubated for 5 min in the dark at 4 °C. Cells were washed and centrifuge at 300×*g* for 5 min. Cell pellets were again resuspended in 80 μl of PBS-0.5% BSA buffer per 10^7^ total cells. 20 μl of Anti-Biotin MicroBeads were added and incubated for 10 min in the dark at 4 °C. The volume was adjusted to 500 µl per 10^7^ total cells with PBS-0.5% BSA buffer and then proceed to magnetic separation. The pre-wet LS columns (Miltenyi Biotec, 130-042-401) were placed at QuadroMACS™ Separator (Miltenyi Biotec, 130-090-976). The cell suspensions were applied onto the columns. The columns were washed with 2 × 1 ml PBS-0.5% BSA buffer. The flow-through containing the unlabelled cells were collected as the neuron-enriched fractions. The columns were removed from the magnetic field, and the non-neuronal cells were flushed out using 3 ml of PBS-0.5% BSA buffer [67].

### Isolation of microglia and astrocytes

Microglia and astrocyte isolation via FACS was performed in *n* = 4 *Grn*^*−/−*^ and *n* = 3 WT brains. Prepared single cell pellets were resuspended in 100 μl of cold D-PBS and stained with 1.5 μl of CD11b-VioBlue^®^ (130-113-810, Miltenyi Biotec) and 1.5 µl of ACSA-2- APC-VIO^®^ 770 (130-116-247, Miltenyi Biotec) antibodies and incubated for 20 min in the dark at 4 °C. The samples were washed with 1 ml of D-PBS and centrifuged at 400×*g* for 5 min. After aspiration of supernatants the cell pellets were resuspended in 300 μl of D-PBS.

Before sorting 1 ml of D-PBS was added and samples were transferred to FACS tubes. Labelled cells were sorted using a MoFlo Astrios EQ cell sorter (B25982, Beckman Coulter) with a threshold set to 30,000 cells for microglia and 100,000 cells for astrocytes, twice negative cells representing the depleted fraction.

### Gamma emission measurements

Radioactivity concentrations of cell pellets were measured in a highly sensitive gamma counter (Hidex AMG Automatic Gamma Counter, Mainz Germany) relative to the activity in the whole brain, with decay-correction to time of tracer injection for final activity calculations. Cell numbers were determined via flow cytometry using a MACSQuant10™

### Immunoblotting and immunohistochemistry

Mice were sacrificed by CO_2_ inhalation or by deep/lethal anaesthesia and perfused with cold PBS. Brain tissue was dissected from adult mice and was snap frozen in liquid nitrogen, mechanically pulverized and stored at − 80 °C for biochemical analysis.

Brain powder were lysed in NP-40 STEN-lysis buffer [23] (150 mM NaCl, 50 mM Tris–HCl pH 7.6, 2.5 mM ETDA, 1% NP40) supplemented with protease inhibitor cocktail (# P8340, Sigma-Aldrich). Lysates were centrifuged for 20 min, 17,000×*g*, 4 °C. The protein concentration of the supernatant was determined using the BCA protein assay (Pierce, Thermo Fisher Scientific) and 20 µg of protein were separated by SDS–PAGE, transferred onto polyvinylidene difluoride membranes (Immobilon-P, Merck Millipore). Membranes were blocked for 1 h in I-BlockTM (# T2015, Thermo Fisher Scientific). Proteins of interest (Table 2) were detected by the indicated primary antibodies followed by horseradish peroxidase-conjugated secondary antibodies detected by ECL Plus (# 32132X3, PierceTM ECL Plus Western Blotting Substrate, Thermo Fisher Scientific). For the quantitative analysis, images were taken by a Luminescent Image Analyzer LAS-4000 (Fujifilm Life Science, Tokyo, Japan) and evaluated with the Multi GaugeV3.0 software (Fujifilm Life Science, Tokyo, Japan). Iba1 immunohistochemistry was performed as described previously [67].

**Table 2:** Antibodies for immunoblotting

Mouse anti-β-actin	1:5000	# A 5316, Sigma-Aldrich
Mouse anti-CD11b/Integrin alpha M	1:3000	# MAB11241, R&D Systems
Mouse anti-Glutamine synthetase	1:1000	# MAB302, Millipore

### Correlation and connectivity analyses

Metabolic brain connectivity patterns can be derived with various methods, including inter-regional correlation coefficients (ICC) [68]. In this work, we assessed metabolic connectivity ICCs of global mean scaled FDG–PET images as published previously [19, 19]. For each analyzed group an inter-regional correlation matrix resulting in 24 × 24 individual Pearson’s correlation coefficients pairs was created. Fisher’s *R*-to-*Z* transformation was performed for all values to yield normal distribution [28]. Brain regions were assigned to functional brain networks (Fig. 1B): Hippocampus CA3, entorhinal cortex, piriform cortex, hypothalamus, thalamus, and amygdala were bundled into a subcortical network. The visual cortex, auditory cortex, somatic motor cortex, and somatosensory cortex were grouped into a cortical network.

**Fig. 2:**
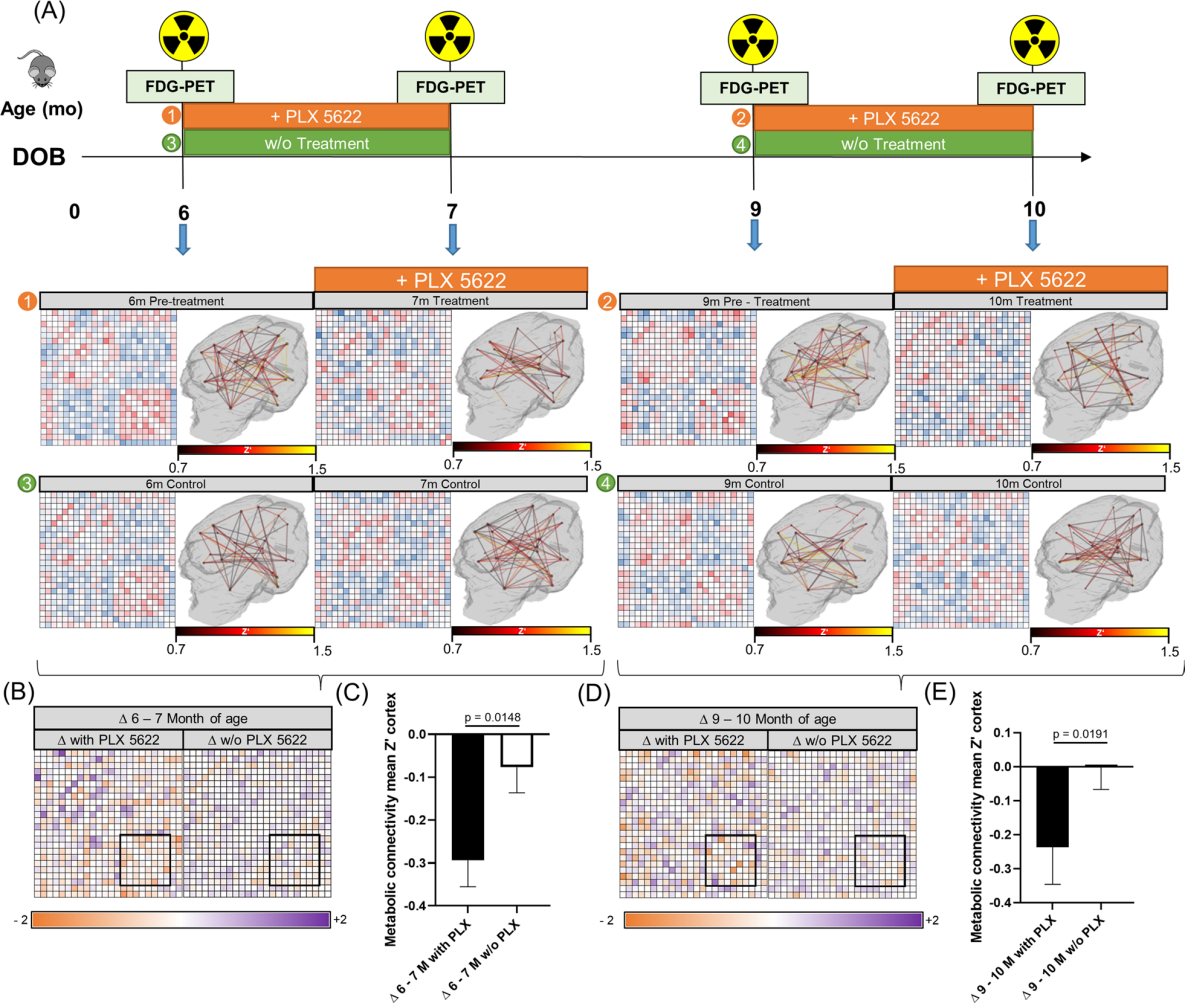
Metabolic connectivity changes in WT mice upon microglial depletion at two different ages. **A** Study overview: two groups of PLX5622 treated WT mice were compared with two groups of age-matched control WT mice. Treated mice received the CSF1R inhibitor for a duration of 5–7 weeks at either 6 months or 9 months of age. The follow-up PET scan was performed under treatment. Single boxes indicate inter-regional *Z*’. All absolute hot-scaled ICCs are presented upon a 3D mouse brain template. **B**, **D** Single matrix elements indicate the difference in inter-regional *Z*’ upon treatment/vehicle. **C**, **E** Bar graphs depict the reduction of average cortical *Z*’ upon microglia depletion. Error bars represent the standard error of the mean. Significance was obtained by a paired *t* test

**Fig. 3:**
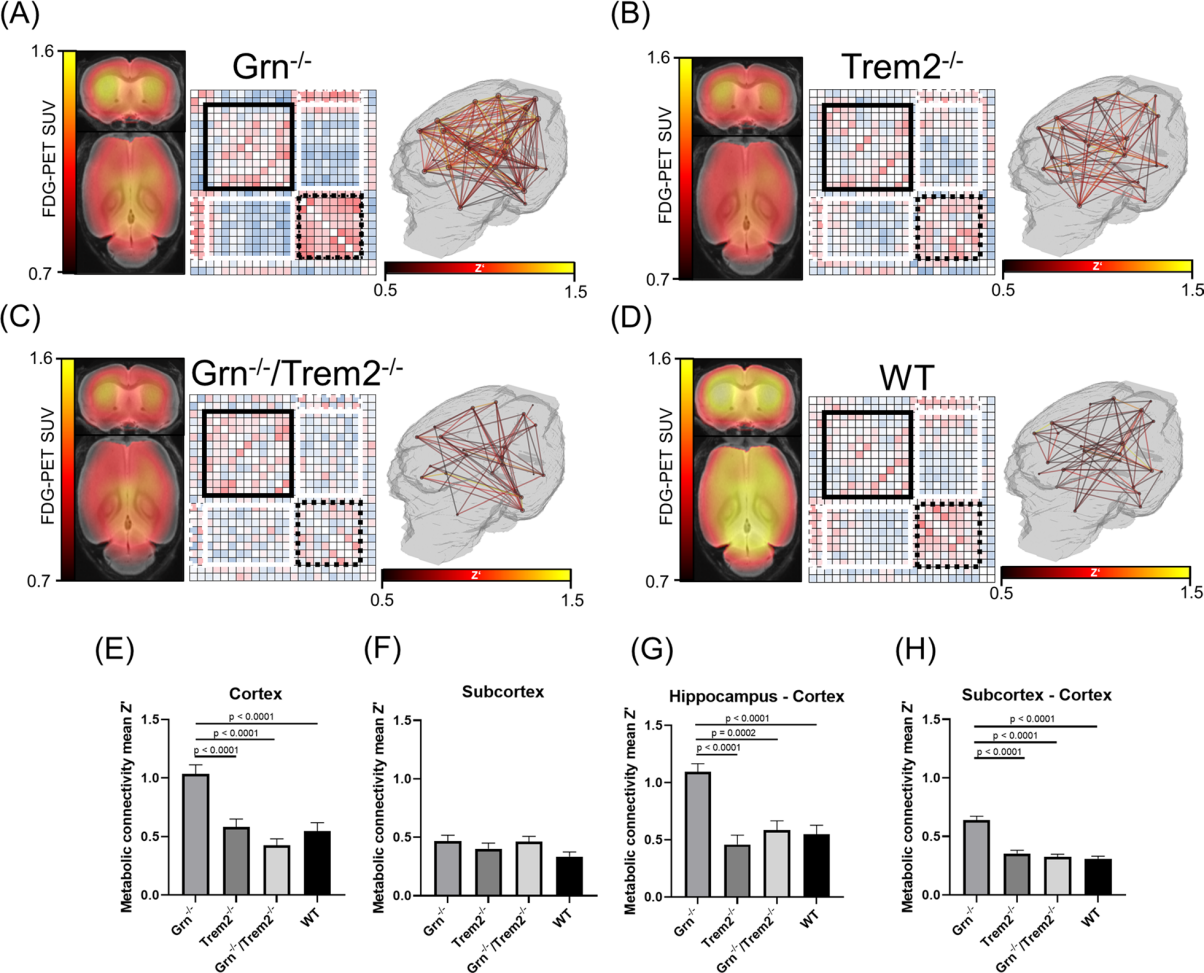
Comparison of metabolic connectivity and FDG–PET SUV in *Grn*^*−/−*^*, Trem2*^*−/−*^*, Grn*^*−/−*^*/Trem2*^*−/−*^ and WT mice. **A**–**D** Hot scaled FDG–PET images of 10.6 ± 1.5-month-old mice depict glucose uptake in axial and coronal images upon a MRI template. Single matrix elements indicate inter-regional *Z*’. Subcortical regions are highlighted with black lines, cortical regions with black dots. Connections between Hippocampus and Cortex as well as Subcortex and Cortex are highlighted with white dots and white lines. All absolute hot scaled ICCs are presented upon a 3D mouse brain template. **E**–**H** Bar graphs indicate mean *Z*’ of the absolute values for each highlighted brain region. Error bars represent the standard error of the mean. Statistics were derived from one-way repeated measures ANOVA with Tukey’s multiple comparisons test

To visualize the highest ICCs in each mouse group, a custom-made Python code based on Matplotlib and Scikit-image was applied. Centers of the VOIs with at least one ICC above a threshold (0.7 for Fig. 2, 0.5 for Fig. 3) were projected into a 3D mouse brain template and displayed as nodes. The node size represents the number of above-threshold ICCs of the corresponding VOI. Connection lines between the nodes display the ICC values between the corresponding VOIs.

### Statistical analyses

Statistical tests were performed using SPSS (V27.0; IBM Corp., Chicago, IL, USA) and GraphPad Prism 8. Differences between ICCs of independent groups were statistically evaluated by one-way ANOVA with Tukey’s post-hoc test. Differences between matched ICCs of two groups were subject to a paired *t* test. When comparing multiple groups, two-way ANOVA was applied, followed by Bonferroni's multiple comparisons tests. A significance level of *p* < 0.05 was applied.

### Data availability

Data and code reported in this article will be shared with any appropriately qualified investigator upon email request.

## Results

### Microglia depletion strongly reduces metabolic connectivity in WT mice

We investigated the effect of microglial depletion on the metabolic connectome by oral administration of a Colony stimulating factor 1 receptor (CSF1R) inhibitor PLX5622 for 7 weeks [67]. Similar to previous work, we observed a 96% reduction of Iba1 immunoreactivity (Additional file 1: Fig. S1).

A strong decrease of ICCs was observed in WT mice treated with PLX5622 when compared to aged-matched vehicle-treated control mice (Fig. 2A, B). Differences in mean cortical ICCs between baseline (pre-treatment) and follow-up (under treatment) were significantly higher in the PLX5622 treated mice when compared to vehicles for both ages studied (6 months; *p* = 0.0148, 9 months; *p* = 0.0191) (Fig. 2C, E. In line with our previous study [67], reduced cortical glucose uptake (SUV) was observed in treated mice at the follow-up timepoint compared to baseline when using the specific cortical VOIs of the current study (Additional file 1: Fig. S2A, B). This reduction was consistent for global mean scaled cortical SUVr quantification (Additional file 1: Fig. S2C, D).

### *Enhanced metabolic connectivity in Grn*^−/−^*mice despite lower FDG–PET signal*

By SUV quantification, we confirmed our previous findings on cerebral hypometabolism in cortical regions of *Grn*^−/−^, *Trem2*^*−/−*^ and *Grn*^*−/−*^*/Trem2*^*−/−*^ [18]. This finding was consistent for SUVr quantification (Additional file 1: Fig. S3).

Either loss of *Grn* or loss of *Trem2* resulted in cerebral hypometabolism [18]. Thus, we tested if metabolic connectivity in *Grn*^*−/−*^ and *Trem2*^*−/−*^ mice changes in the same direction when compared to WT mice. For this purpose, we studied *Grn*^*−/−*^ mice (*n* = 10), *Grn*^*−/−*^*/Trem2*^*−/−*^ mice (*n* = 10), *Trem2*^*−/−*^ mice (*n* = 13) and WT mice (*n* = 17) with FDG–PET at an average age of 10.6 ± 1.5 months.

We observed a strong increase in cortical metabolic connectivity of *Grn*^*−/−*^ mice compared to WT mice (Fig. 3A, D), whereas *Trem2*^*−/−*^ mice did not show a significant change of metabolic connectivity when compared to WT mice (Fig. 3B, D) (ANOVA: *F* value: 15.11, *p* < 0.0001). The metabolic connectivity change of *Grn*^*−/−*^ mice was ameliorated in the double knockout *Grn*^*−/−*^*/Trem2*^*−/−*^ mice when compared to WT mice (Fig. 3C, D). This observation was mirrored for hippocampal networks (*p* = 0.0002, Fig. 3G) and for ICCs between cortical and subcortical compartments (*p* < 0.0001, Fig. 3H). In the subcortical network (Fig. 3F), we did not observe a significant impact of *Grn* and/or *Trem2* deficiency on metabolic connectivity when compared to WT mice.

### *Enhanced metabolic connectivity in Grn*^−/−^* mice is accompanied by a relative shift towards microglial FDG allocation*

**Fig. 4:**
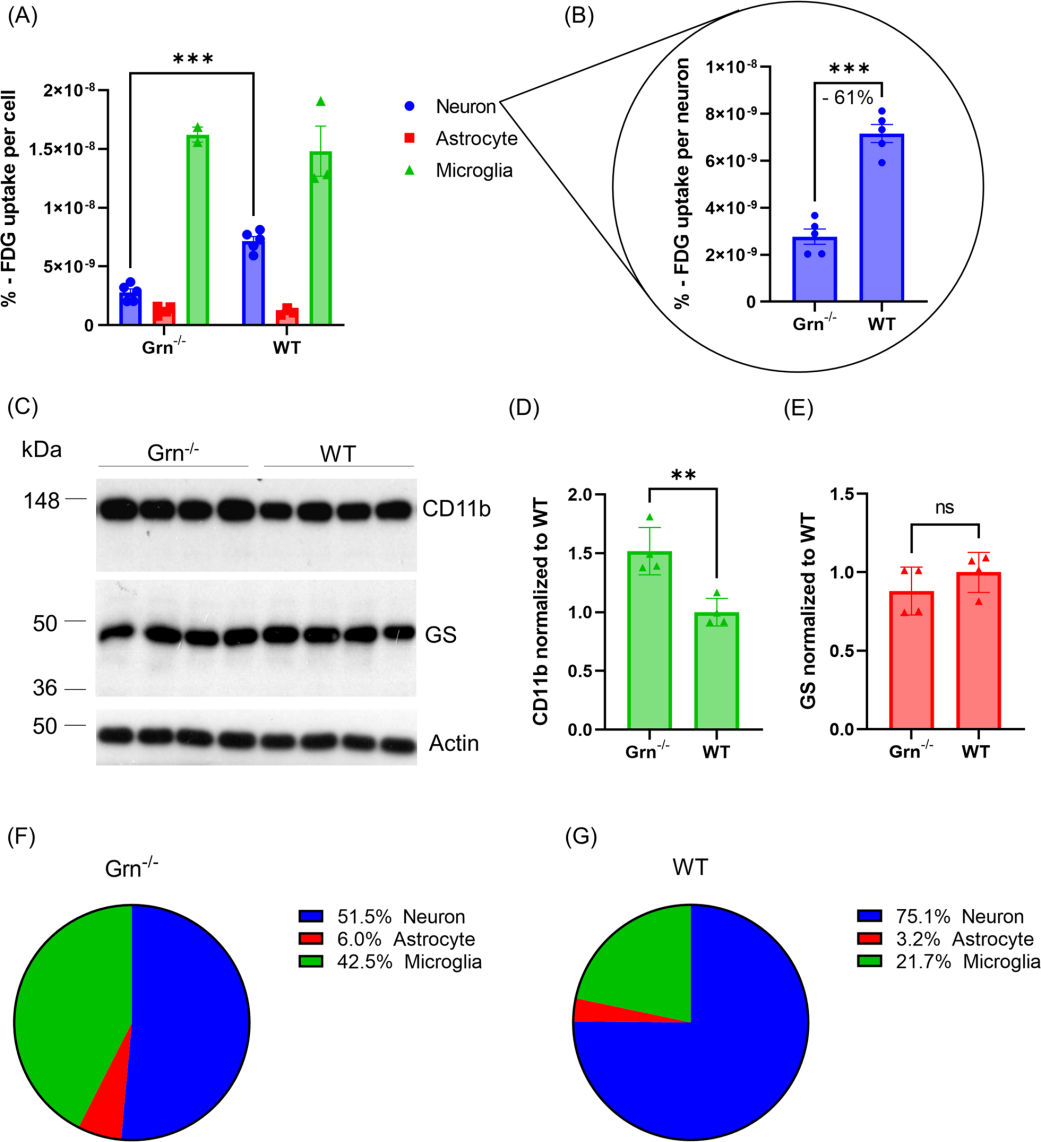
Cellular allocation of FDG uptake in *Grn*^*−/−*^ and WT mice. **A**, **B** %-FDG uptake per cell in the isolated neuron, astrocyte, and microglia fractions from *Grn*^*−/−*^ and WT mice. Mean ± SEM of *n* = 2–5 animals. Two-way ANOVA with Bonferroni correction for multiple comparisons, *Grn*^*−/−*^ neuron vs. WT neuron *p* = 0.0006. **C–E** Immunoblot expression of CD11b, glutamine synthetase and Actin, as a loading control, in *Grn*^*−/−*^ vs. WT, *n* = 4 mice per group. Unpaired *t* test, CD11b *p* = 0.0044; GS *p* = 0.7753. **F**, **G** Relative proportions of brain FDG allocation accounting from neurons, astrocytes, and microglia

Finally, we explored the different cellular contributions to FDG allocation resulting from neuronal, astrocytic, and microglial glucose uptake in *Grn*^*−/−*^ and WT mice. For this purpose, we isolated cells after FDG-injection via MACS and FACS scRadiotracing for gamma emission measures [5]. As expected from previous findings [67], microglia showed the highest FDG uptake per cell in WT mice (1.48e^−8^% ± 3.70e^−9^%). Microglia (1.62e^−8^% ± 9.00e^−10^%) and astrocytes (1.27e^−9^% ± 3.79e^−10^%) of *Grn*^*−/−*^ mice indicated a similar %-FDG uptake per cell (microglia + 9%, *p* = 0.92,astrocytes + 12%, *p* = 0.99) compared to WT (Fig. 4A). Strikingly, neurons of *Grn*^*−/−*^ mice (2.77e^−9^% ± 7.24e^−10^%) had a severe reduction of FDG-uptake per cell when compared to neurons of WT mice (7.16e^−9^% ± 8.60e^−10^%, − 61%, *p* = 0.0006) (Fig. 4A, B). Immunoblot of CD11b and glutamine synthetase (GS) in eight mice, with an average age of 10.8 ± 1.5 months, confirmed higher microglial abundance (+ 52%, *p* = 0.0044) but no significant expression changes of the astrocyte marker GS (− 12%, *p* = 0.7753) in *Grn*^*−/−*^ mice when compared to WT mice (Fig. 4C–E). The combined observations resulted in a shift of relative FDG allocation towards the microglia fraction (42% vs. 22%; Fig. 4F, G).

## Discussion

In this study, we investigated metabolic connectivity upon pharmacological depletion of microglia and in mouse models with distinct microglia phenotypes. Microglia depletion demonstrated that microglial glucose uptake has a significant contribution to the metabolic connectome of the mouse brain. *Grn*^*−/−*^ mice with hyperactive microglia revealed a strong increase of metabolic connectivity in cortical networks despite significantly lower FDG–PET signal. Importantly, the strong reduction of neuronal glucose uptake with a shift of glucose allocation to the microglia fraction was accompanied by enhanced metabolic connectivity.

Thus, our findings highlight the impact of microglial glucose uptake on metabolic connectivity.

Since PET studies do not distinguish the cellular site of glucose metabolism, inflammatory cells could mask metabolic deficits in neurodegenerative disease by increased glucose consumption even in brain areas with insufficient supply for neurons and astrocytes to survive [3]. However, the total FDG uptake measured by PET is consequently the composite of neuronal, astroglial [70] and microglial cells [3, 8, 67]. Our previous study provided evidence that the FDG–PET signal is primarily influenced by microglial glucose uptake [67]. In mouse models with amyloidosis, the increased FDG–PET signal was mainly driven by activated microglia and was entirely eliminated upon PLX5622-induced microglia depletion [67]. Current data confirmed the reduction of FDG–PET signals in WT mice upon microglia depletion at different ages. Our first main finding indicates that treatment with a CSF1R inhibitor in WT mice leads to a significant reduction of metabolic connectivity when compared to vehicle-treated age-matched littermates. CSF1R is essential for microglial environment and viability [11], and we confirmed by immunostaining that 96% of the cortical microglia population were depleted after 7 weeks of treatment (Additional file 1: Fig. S1) [67]. Sufficient removal of microglia by CSF1R inhibition was also previously shown in mouse models of Parkinson’s disease [45], 4-repeat tauopathies [47] and during traumatic brain injury [26]. In this regard, it remains unclear whether microglia are themself responsible for consumption of their high glucose uptake or if the energy utilization takes place in other cells. Although, upregulation of glucose 1 transporters in activated microglia was recently shown [64], presence of mechanisms such as astrocyte–neuron lactate shuttle [49] and oligodendrocyte–neuron lactate shuttle [16, 34] could also be present as microglia–neuron shuttle and deserve further investigation.

Taken together, our previous findings on microglial activation driving FDG–PET alterations [67], and our current results on the impact of microglial absence and activation on metabolic networks, interrogate neurons as the main source of metabolic connectivity and also question the term “metabolic” connectivity, which may better be termed “uptake” connectivity.

Numerous genes expressed in microglia are associated with elevated risk for neurodegenerative diseases, such as AD [21, 35] and FTD [4, 18, 32, 53, 66]. Previously, we demonstrated opposite microglia phenotypes resulting from loss of *Grn* and *Trem2*. In line with previous data, FDG–PET signals indicated a reduction of FDG-uptake in presence of hyperactive microglia (*Grn*^*−/−*^) and microglia that is locked in a homeostatic state (*Trem2*^*−/−*^) [18, 32, 40, 53]. This finding did not directly fit to the previously observed linkage between microglial activation states and higher glucose uptake [67]. *Trem2* deficient mice not only showed sustained but even increased expression of homeostatic genes compared to WT mice, leading to impaired activation and, therefore, locking mice in a resting state impairing a potential protective response in neurodegenerative conditions [40]. Thus, we compared the metabolic connectivity and single-cell FDG uptake in *Grn*^*−/−*^ and WT mice, and we compared metabolic connectivity of *Grn*^*−/−*^ mice against *Trem2*^*−/−*^ mice and the *Grn*^*−/−*^*/Trem2*^*−/−*^ double knockout mice. Next, we questioned if the activation status of microglia has an impact on metabolic connectivity in *Grn*^*−/−*^ mice and indeed we observed a striking elevation of metabolic connectivity when compared to WT mice. Importantly, scRadiotracing revealed a severe reduction of neuronal FDG uptake but similar microglial and astroglial FDG uptake when compared to WT mice. In addition, to the results of scRadiotracing, we observed a significant elevation of CD11b expression in *Grn*^*−/−*^ compared to WT mice. In conjunction with our previously published data which state unchanged expression of CD11b per single microglial cell in *Grn*^*−/−*^ mice [53, 66] our data indicates proliferation with a 1.5 fold higher abundance of microglia in *Grn*^*−/−*^ mice. By use of a CD11b antibody, which is not entirely specific for microglia, we also accounted for a minor proportion of CD11b positive macrophages and invading peripheral cells in the brain, which may have a limited impact on our results. We intentionally omitted to perfuse the mice for cell isolation to resemble the in vivo tracer allocation as established previously for comparability reasons [67]. In conjunction with our previous data [53, 67], it appears obvious that the reduced FDG–PET signal in *Grn*^*−/−*^ mice is driven by reduced FDG uptake of neurons, whereas the reduced FDG–PET signal in *Trem2*^*−/−*^ mice is driven by reduced FDG uptake of microglia. In line, several studies using *Grn*^*−/−*^ mice demonstrated reduced synaptic connectivity, impaired synaptic plasticity, decreased dendritic length and spine density (Petkau et al. [51], decreased neuronal activation with unchanged total number of neurons in the amygdala [12] and absent neuronal loss in the thalamus [1]. Given these data, we considered neuronal cell numbers to be unchanged between *Grn*^*−/−*^ and WT mice. It is most likely that that deficiency in Grn^−^/^−^ mice also specifically affects neurons and their glucose metabolism, leading to the observed decreased glucose uptake. In this regard, contrary effects in neurons to the previously shown upregulation of microglial glucose transporter in the presence of inflammatory conditions [64] are conceivable. Further studies could elucidate this hypothesis in Grn deficient neurons by correlating scRadiotracing with their glucose transporter expression and mitochondrial metabolism, glycolysis and oxygen consumption rate using the Seahorse Extracellular Flux Analyzer.

However, we note that some previous studies showed that microglia lacking *Grn* were more cytotoxic than WT microglia leading to hippocampal cell death with increased vulnerability of neurons to stress and inflammatory brain injury [69] and to increased neuronal death due to alterations in secreted factors by *Grn* deficient microglia [38]. Thus, the shift towards higher relative microglial allocation of glucose uptake could be even higher, but given the aforementioned controversies in literature, we decided on the conservative variant. Nevertheless, we also observed a significant increase of total FDG-uptake enhancement in *Grn*^*−/−*^ mice by astrocytes, which was proportionally similar to the microglial uptake. In line with previous published data, our results indicate that at the age of 10.8 ± 1.5 months, microgliosis is apparent in *Grn*^*−/−*^ mice, while astrocyte proliferation has not yet launched at this stage [1]. Besides the aforementioned upregulation of glucose transporters in activated microglia [64], also astrocytes adapt to proinflammatory environment with modified metabolic phenotypes increasing their glucose utilization [17]. Perhaps the affected neuronal glucose uptake in *Grn* deficient mice induces compensatory enhanced astrocyte glucose transporter expression to provide substrates for astrocyte–neuron lactate shuttles [39] or glycogenolysis [9], to maintain neuronal energy supply. In total, however, the proportion of astrocyte FDG-uptake in WT and *Grn*^*−/−*^ mice was little. In synopsis with the significantly altered metabolic connectivity in microglia but not astrocyte depleted mice, the impact of astrocytes on metabolic connectivity might be negligible.

The metabolic connectome was strongly altered in *Grn*^*−/−*^ mice, but unchanged in *Trem2*^*−/−*^ and *Grn*^*−/−*^*/Trem2*^*−/−*^ mice when compared to WT mice. Fitting to this observation, we observed lower single-cell FDG uptake of microglia in *Trem2*^*−/−*^ mice [67]. As a limitation, we note that we did not perform scRadiotracing in *Grn*^*−/−*^*/Trem2*^*−/−*^ mice. Here, the simultaneous knockout of *Grn*^*−/−*^ and *Trem2*^*−/−*^ [53], characterized by a reduced cerebral FDG–PET signal, showed amelioration of metabolic network changes observed in *Grn*^*−/−*^ mice, though neurotoxicity was not rescued [53]. Indeed, the lacking rescue of neurotoxicity with striking increase of neurofilament light chain (NfL) in the cerebrospinal fluid (CSF) of *Grn*^*−/−*^ /*Trem2*^*−/−*^ mice [53] could explain the metabolic network changes, which may be a composite result of simultaneous reductions in neuronal and microglia glucose uptake.

We further note that the disparity between female and male mice included and analyzed in our study needs to be acknowledged. Previous studies have explored sex differences of microglia in humans and rodents [24, 63], considering microglial phenotype [22] cell numbers [43], and development [25] but also their effect on TSPO–PET binding [6]. Given our increasing understanding of these important variances of sex-dependent microglial activation, also distinct metabolic connectivity patterns for both sexes are conceivable. Therefore, further investigations should decipher the impact of sex-dependent microglial activation to metabolic connectivity in mouse models of neurodegenerative diseases. Moreover, although standardized in our study to the best of our knowledge, inter-individual confounds such as general motoric activity, pre-scanning anesthesia duration, diet, age-dependent changes, or cohort size might influence metabolic connectivity patterns.

## Conclusion

In summary, microglial glucose uptake has an essential impact on metabolic connectivity, since pharmacological depletion of microglia results in decreased brain metabolic networks. Microglial hyperactivation in conjunction with a shift of relative FDG uptake in brain towards microglia is associated with strong increases of metabolic connectivity. Thus, FDG uptake derived connectivity measures have the potential to act as a complementary tool to investigate pathological alterations of FDG–PET in mouse models with distinct microglia phenotypes.

## Supplementary Information

**Additional file 1:** **Fig. S1 (A, B)** Representative immunostaining images of the microglia marker Iba1 in the cortex and hippocampus. **(C)** Microglia abundance in the cortex and hippocampus. Mean ± SD of *n* = 3 per group. Significance levels were obtained by an unpaired *t* test. **Fig. S2. (A–D)** Scatter plots display single SUV (**A, B**) and SUVr (**C, D**) values before and after microglia depletion. Error bars indicate standard deviation. Significance was obtained by an unpaired *t* test. **Fig. S3. (A, B)** Scatter plots display single SUV **(A)** and SUVr **(B)** values. Error bars indicate standard deviation. Statistics were derived from one-way ANOVA with Tukey’s multiple comparisons test.

## Data Availability

The raw data supporting the conclusions of this article will be shared with any appropriately qualified investigator upon reasonable request.

## Abbreviations

AD: Alzheimer’s disease
ANOVA: Analysis of variance
BSA: Bovine serum albumin
CA1: Cornu ammonis 1
CA3: Cornu ammonis 3
CSF: Cerebrospinal fluid
CSF1R: Colony stimulating factor 1 receptor
Ctx: Cortex
DAM: Disease-associated microglia
DLB: Dementia with Lewy bodies
FACS: Fluorescence activated cell sorting
FDG: Fluordesoxyglucose
fMRI: Functional magnetic resonance imaging
FTD: Frontotemporal dementia
Grn: Progranulin
GS: Glutamine synthetase
ICC: Interregional correlation coefficients
MACS: Magnetic activated cell sorting
NfL: Neurofilament light chain
OSEM: Ordered subset expectation maximization
PBS: Phosphate-buffered saline
PET: Positron emission tomography
SEM: Standard error of the mean
SUV: Standardized uptake value
SUVr: Standardized uptake value ratio
Trem2: Triggering receptor expressed on myeloid cells 2
VOI: Volume of interest
WT: Wild type
