# Supplementary material for: Depletion and activation of microglia impact metabolic connectivity of the mouse brain
Source: J Neuroinflammation. 2023 Feb 24;20:47. doi: 10.1186/s12974-023-02735-8 (PMC9951492; doi:10.1186/s12974-023-02735-8)
Supplement: Supplementary file 1 — Additional file 1: Fig. S1 (A, B) Representative immunostaining images of the microglia marker Iba1 in the cortex and hippocampus. (C) Microglia abundance in the cortex and hippocampus. Mean ± SD of n = 3 per group. Significance levels were obtained by an unpaired t test. Fig. S2. (A–D) Scatter plots display single SUV (A, B) and SUVr (C, D) values before and after microglia depletion. Error bars indicate standard deviation. Significance was obtained by an unpaired t test. Fig. S3. (A, B) Scatter plots display single SUV (A) and SUVr (B) values. Error bars indicate standard deviation. Statistics were derived from one-way ANOVA with Tukey’s multiple comparisons test. [file 12974_2023_2735_MOESM1_ESM.docx]

Additional file 1
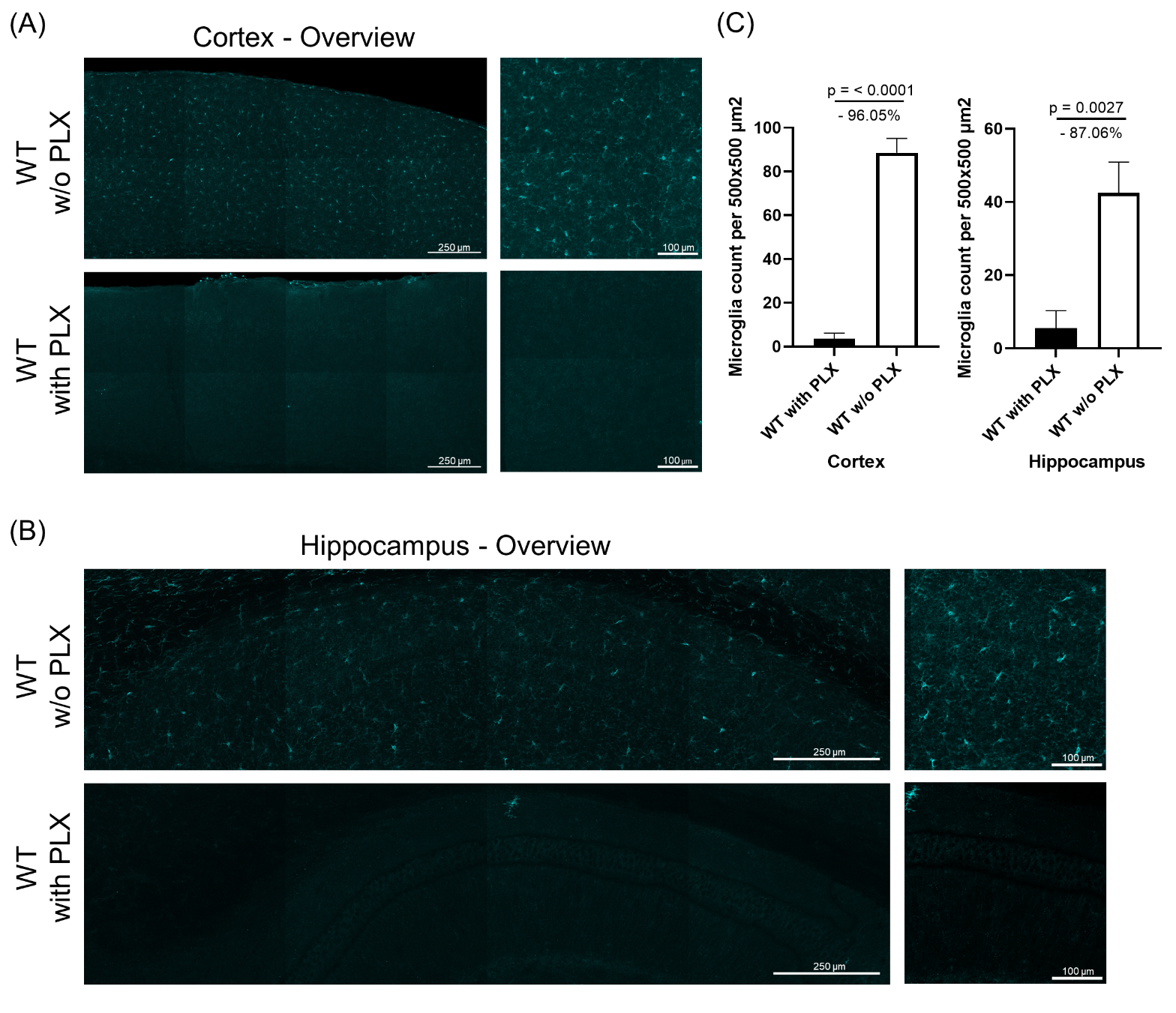


**Fig. S1** **(A-B)** Representative immunostaining images of the microglia marker Iba1 in the cortex and hippocampus. **(C)** Microglia abundance in the cortex and hippocampus. Mean ± SD of n = 3 per group. Significance levels were obtained by an unpaired t-Test.


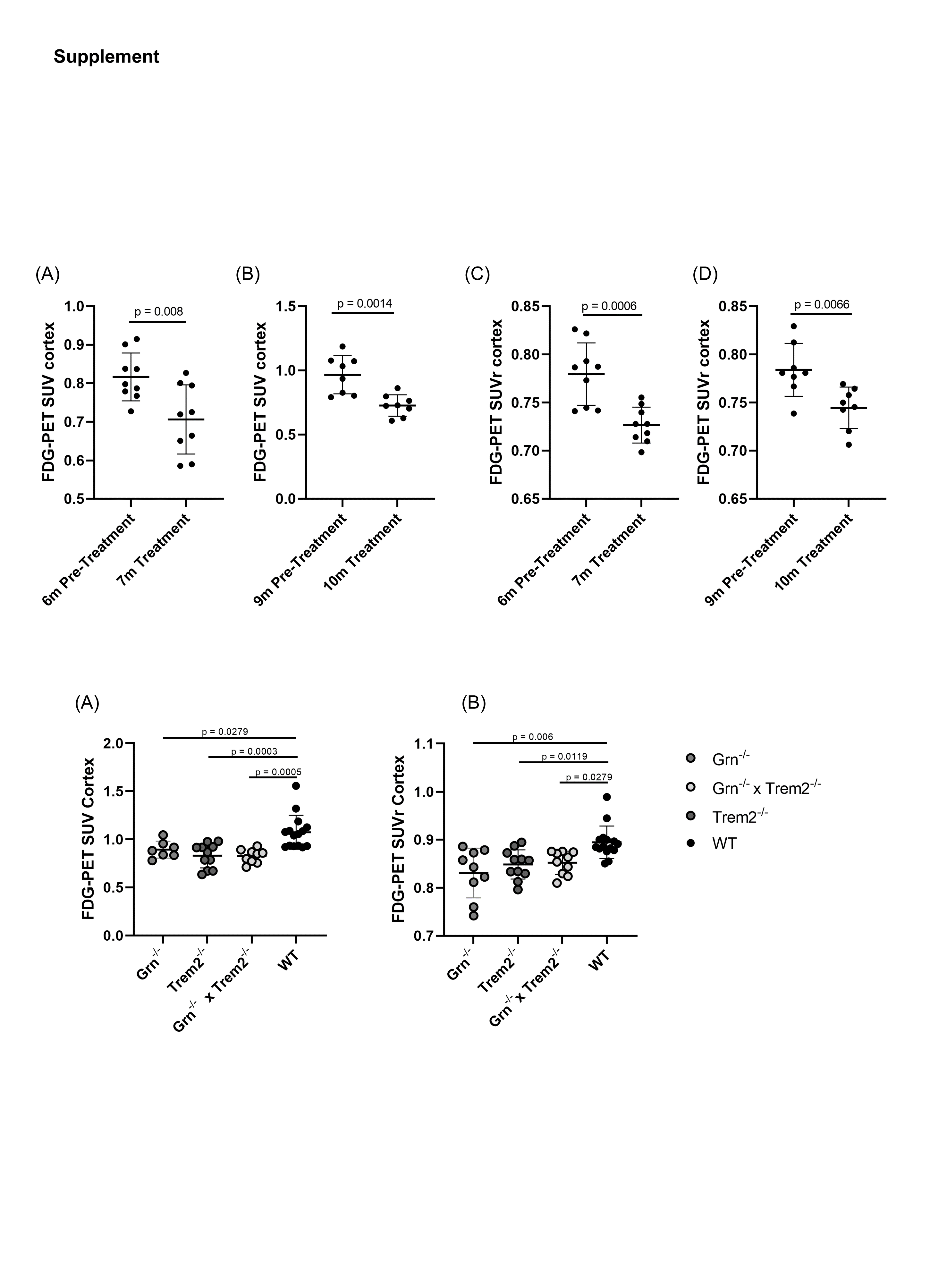


**Fig. S2. (A-D)** Scatter plots display single SUV (**A-B**) and SUVr (**C-D**) values before and after microglia depletion. Error bars indicate standard deviation. Significance was obtained by an unpaired t-test.


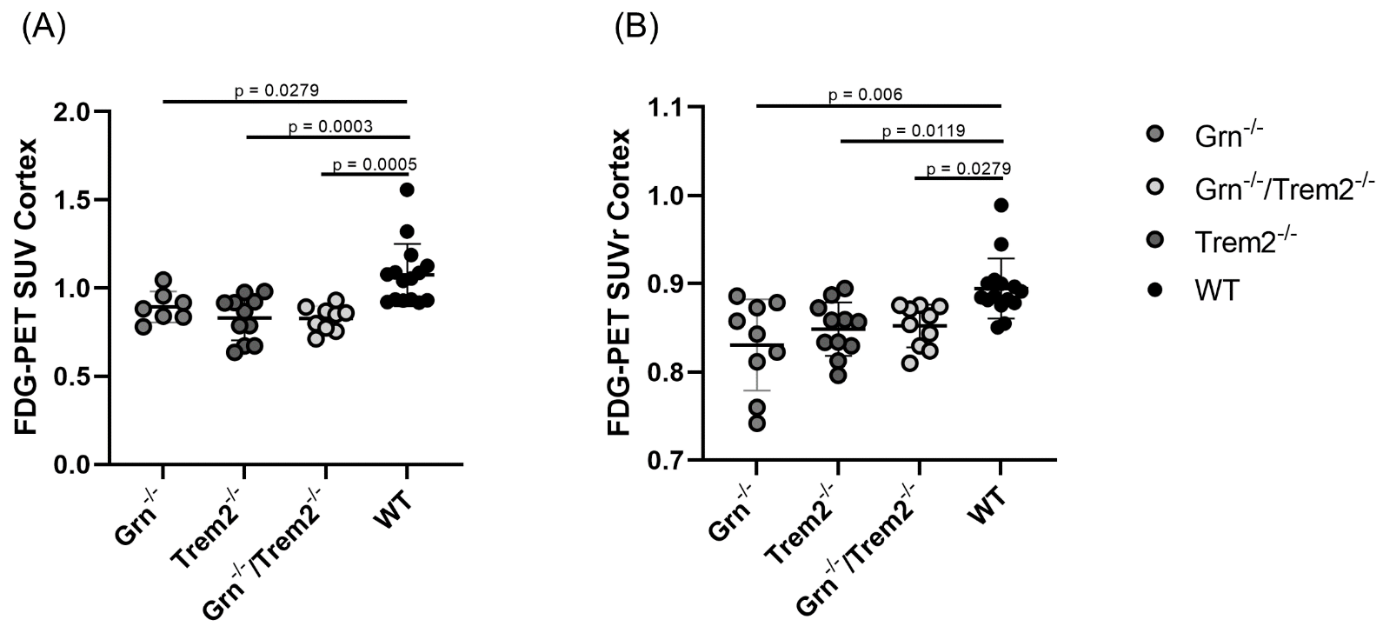


**Fig. S3. (A-B)** Scatter plots display single SUV **(A)** and SUVr **(B)** values. Error bars indicate standard deviation. Statistics were derived from one-way ANOVA with Tukey’s multiple comparisons test.
